# Characterization and Comparative Genomic Analysis of a Highly Colistin-Resistant *Chryseobacterium gallinarum*: a Rare, Uncommon Pathogen

**DOI:** 10.3389/fcimb.2022.933006

**Published:** 2022-07-14

**Authors:** Mahendra Gaur, Suchanda Dey, Anshuman Sahu, Sangita Dixit, S. Sarathbabu, John Zothanzama, Rajesh Kumar Sahoo, Dibyajyoti Uttameswar Behera, Monika, Enketeswara Subudhi

**Affiliations:** ^1^ Department of Biotechnology, Punjabi University, Patiala, India; ^2^ Centre for Biotechnology, School of Pharmaceutical Sciences, Siksha ‘O’ Anusandhan (Deemed to be University), Bhubaneswar, India; ^3^ Department of Biotechnology, Mizoram University, Aizawl, India; ^4^ Department of Biotechnology, Mata Gujri College (Autonomous), Fatehgarh Sahib, India

**Keywords:** *Chryseobacterium gallinarum*, colistin-resistant, capsular polysaccharide (CPS), β-lactamases, uncommon pathogen, α-hemolysis, comparative genomics

## Abstract

For the first time, we describe the whole genome of a yellow-pigmented, capsule-producing, pathogenic, and colistin-resistant *Chryseobacterium gallinarum* strain MGC42 isolated from a patient with urinary tract infection in India. VITEK 2 automated system initially identified this isolate as *C. indologenes*. However, 16S rRNA gene sequencing revealed that MGC42 shared 99.67% sequence identity with *C. gallinarum*–type strain DSM 27622. The draft genome of the strain MGC42 was 4,455,926 bp long with 37.08% Guanine-Cytosine (GC) content and was devoid of any plasmid. Antibiotic resistance, virulence, and toxin genes were predicted by implementing a machine learning classifier. Potential homologs of 340 virulence genes including hemolysin secretion protein D, metalloprotease, catalase peroxidases and autotransporter adhesins, type VI secretion system (T6SS) spike proteins, and 27 toxin factors including a novel toxin domain Ntox23 were identified in the genome. Kyoto Encyclopedia of Genes and Genomes (KEGG) orthologs of 110 transporter proteins were predicted that were in agreement with moderate efflux activity. Twelve antibiotic resistance genes including two potentially novel putative β-lactamase genes sharing low similarity with known β-lactamase genes were also identified in the genome of this strain. The strain MGC42 was also resistant to several classes of antibiotics along with carbapenems and polymyxin. We also identified mutations in the orthologs of *pmr*B (M384T) and *lpx*D (I66V) that might be responsible for colistin resistance. The MGC42 strain shared 683 core genes with other environmental and clinical strains of *Chryseobacterium* species. Our findings suggest that the strain MGC42 is a multidrug-resistant, virulent pathogen and recommend 16S rRNA gene sequencing to identify clinical specimens of *Chryseobacterium* species.

## Introduction

The frequency of healthcare-associated infections caused by rare or uncommon pathogens like *Chryseobacterium* species has risen over the last decade (Booth, 2014). The high rate of infection of these bacterial pathogens attracts attention for proper management as these are inherently resistance to aminoglycosides, aztreonam, cephalosporin, chloramphenicol, clindamycin, erythromycin, imipenem, penicillin (mezlocillin, piperacillin, and ticarcillin), teicoplanin, and tetracyclines (Hsueh et al., 1997; Sharma et al., 2015). These are chemoorganotrophic, glucose-nonfermenting, non-motile, rod-shaped gram-negative, and emerging clinical pathogenic bacteria ubiquitously detected in soil and water (Hugo et al., 2019). Recently, *C. gleum* and *C. oranimense* are reported as the pathogenic species and linked to ventilator-associated pneumonia, urinary tract infection (UTI) (Rajendran et al., 2016), and cystic fibrosis (Sharma et al., 2015). However, their genetic basis of resistance mechanisms, pathogenicity, and virulence is still poorly known.

Among the *Chryseobacterium* sp., *C. indologenes* has been identified as opportunistic pathogens to nosocomial infections in immunocompromised patients of all ages (Reynaud et al., 2007; Smith et al., 2012). It has been well documented in a variety of illnesses (nosocomial pneumonia, intra-abdominal infection, wound infection, bacteremia, UTI, and cellulitis), particularly in those who were hospitalized with long-term indwelling devices and were exposed to broad-spectrum antibiotics for an extended period (Christakis et al., 2005; Chen et al., 2013). Multidrug resistance *in C. indologenes* has been reported due to increased clinical usage of colistin and tigecycline, which poses a concern to patients who had received substantial antibiotic therapy for an extended period (Chen et al., 2013). Although the source of this infection is unknown, the occurrence of MDR *C. indologenes* has been well documented in seawater and marine fauna (Maravić et al., 2013).

This study aims to describe the whole-genome sequence used to insight the resistome, virulome, and toxic profile of colistin-resistant *C. gallinarum*, isolated, for the first time, from the urine of a female patient diagnosed with UTI in Bhubaneswar city at our university’s tertiary care hospital. It indicated that *C. gallinarum* might have the potential to grow in uroepithelial cells. This species was first isolated in 2014 from a pharyngeal scrape of a healthy chicken in Germany and showed keratin degrading activity (Kämpfer et al., 2014). Previously, Park et al. (2015) and Kang et al. (2021) have provided genetic insight into the keratin degradation mechanism in this species. We also explored several unique features, i.e., oxidative-stress response, hemolysis activity, and capsular polysaccharide (CPS) secretion ability. We evaluated the shared conserved genes and their potential role in different habitats, including natural and clinical environments through comparative genomics analysis by incorporating genomes of other *Chryseobacterium* species.

## Methodology

### Sample Collection, Identification, and Antimicrobial Susceptibility

During a surveillance study conducted during the period of 2018–2019, a colistin-resistant bacteria MGC42 was recovered in Bhubaneswar city at Central laboratory of our university’s tertiary care hospital from a 20-year old Outpatient Department (OPD) patient diagnosed with UTI. We initially identified the organism and subsequently tested its antimicrobial susceptibility with the VITEK 2 automated system (BioMérieux, France) using the ID-GNB and AST-381 cards, respectively, in accordance with the manufacturer’s instructions. We interpreted the results of antibiotic susceptibility based on the Clinical and Laboratory Standards Institute (Wayne, 2018) breakpoint recommendations. The identity of the strain MGC42 was further verified by amplification and sequencing its 16S rRNA gene using 16S rRNA universal primers (16S-F, 5′-AGAGTTTGATCATGGCTC-3′; 16S-R, 5′-GGTTAC CTTGTTACGACTT-3′). The 16S rRNA gene sequence was then searched using BLAST (https://blast.ncbi.nlm.nih.gov/Blast.cgi) and compared with other 16S rRNA sequences available in GeneBank of National Center for Biotechnology Information (NCBI). To determine whether the cells produced flexirubin-type pigments, we flooded a mass of bacterial cells collected on a LB agar plate with 20% (w/v) KOH. The mass instantaneously turns dark red/brown (Reichenbach, 1989) if cells produce flexirubin-type pigments, whereas no color change develops if the yellow color is not due to carotenoid type of pigments. Minimum inhibitory concentrations (MIC) values for colistin and meropenem were determined by the broth micro-dilution method using cation-adjusted Mueller–Hinton broth according to the Clinical and Laboratory Standards Institute (CLSI) guidelines (CLSI, 2018). The lowest concentration of antibiotics that completely inhibited microbial growth was considered MIC.

### DNA Isolation, Whole-Genome Sequencing, Annotation, and Comparative Genomics

We extracted the genomic DNA of the strain by using a modified ROSE (rapid one-step extraction) method (Dey et al., 2020). Briefly, the bacterial cells were harvested at mid-log phase, and pellet was incubated with 500 µl of ROSE solution for 60 min at 90°C with intermittent shaking. Phenol, chloroform, and isoamyl alcohol were subjected at the ratio of 25:24:1. The aqueous layer was separated upon centrifugation at 12,000 × g for 30 min. Precipitation of the sample was done with 2.5 volumes of ethanol at −20°C. DNA pellet was dissolved in 100 µl of T10E1 (pH 8.0) and treated with RNase (10 gm/ml) at 37°C. The extracted DNA was visualized on 0.8% ethidium bromide-stained agarose gel. We outsourced the sequencing of the genome of *Chryseobacterium gallinarum* (MGC42) to Agrigenome, India, where its genome was sequenced on the Illumina HiSeq platform. The NEBNext Ultra DNA Library Kit was used for library preparation by using 100 ng of total DNA. The sequenced pair-end reads were check for qualitative and quantitative analysis using FastQC v.0.11.5 (Andrews, 2010). Adapters were removed, and low-quality ends were trimmed from the sequences with a sliding window of 4 and a minimum quality of 20 using Trimmomatic v0.36 (Bolger et al., 2014). Then, we *de novo* assembled the quality-filtered fastq reads with Unicycler v0.5.0 assembler (Wick et al., 2017). We further rearranged, reconstructed, and scaffolded the assembled genome into lesser number of contigs using Ragout (Reference-Assisted Genome Ordering UTility) tool based on reference genome of *C. gallinarum* DSM 27622 (GCA_001021975.1) and FDAARGOS_636 (GCA_012273615.1) (Kolmogorov et al., 2014). Finally, we assessed the quality of the genome with QUAST v**5.1 (** Mikheenko et al., 2018 **)** tool and compared our genome with the complete genomes of *C. gallinarum* DSM 27622 (GCA_001021975.1) and FDAARGOS_636 (GCA_012273615.1), respectively.

We predicted the Open Reading Frame (ORFs) and annotated our assembled genome using Prokka (Seemann, 2014) of contigs with length ≥200 bp. We identified the biological pathways and molecular functions of the predicted ORFs using the kofamKOALA (Aramaki et al., 2020). We then identified the antibiotic resistance genes by using the CARD, ARG-ANNOT, ResFinder, and NCBI AMR databases. By implanting k-mer (PATRIC), machine learning, and Hidden Markov models (HMM)-based classifier (PathoFact) (de Nies et al., 2021), we further improved the antibiotic resistance, virulence, and toxin gene annotation. We identified phage sequences in the genome using PHASTER (PHAge Search Tool Enhanced Release) (Arndt et al., 2016). We submitted our genome assembly to the TYGS online server (Meier-Kolthoff and Göker, 2019) for whole-genome–based taxonomic analysis and determination of closest type strain genomes. A cladogram was then inferred on the basis of Genome BLAST Distance Phylogeny approach (GBDP) and visualized in FigTree (https://github.com/rambaut/figtree).

We compared the complete genome of *C. gallinarum* MGC42 to the complete genome of *C. gallinarum* DSM 27622, *C. gallinarum* FDAARGOS_636, *C. contaminans* DSM 27621, *C. oranimense* G311, and *C. indologenes* MARS15 in terms of their shared orthologous, core, and accessory genes using OrthoVenn (Xu et al., 2019) and Roary (Page et al., 2015). We identified and located the O-antigen cluster based on homology with o-antigen cluster of *C. oranimense* G311, *C. gelum* ATCC 35910, and *Chryseobacterium* spp. CF314 (Sharma et al., 2015).

### Characterization of Virulence-Associated Phenotypes

We studied the external morphology of *Chryseobacterium gallinarum* MGC42 using transmission electron microscopy (TEM). We incubated the strain MGC42 overnight in Luria Bertini (LB) broth and then diluted this culture to 5 × 10^6^ cells (OD 0.05) in tryptic soy broth. We loaded 10 µl of culture into electron microscopy grids and dried them under light exposure. We soaked the spare culture in tissue paper. We finally added 2% of caesium chloride to this dried culture and dried it again under light exposure. Finally, we performed TEM at an operating voltage of 200 kV and visualized the images at magnification of ×14,500. We used the ethidium bromide (EtBr) cartwheel method as described by Martins et al., 2011 to determine the Efflux pump activity of MGC42. We used *E. coli* ATCC 25922 and *K. pneumoniae* SDL79 (Dey et al., 2020) as reference and positive control, respectively. We poured Tryptic Soy Agar nutrient media with a range of EtBr concentrations (0–5 μg/ml) in Petri plates and streaked the overnight culture of these strains in a cartwheel pattern on the plates. The plates were then incubated overnight at 37°C and observed under UV for fluorescence (Bio-Rad Gel-Doc XR system, Hercules, CA, USA). We validated the presence of functional catalases by bubble test (catalase test) using the slide method (Reiner, 2013; Kataria et al., 2016). We added about three to four drops of 3% medical hydrogen peroxide (H_2_O_2_) to the fresh culture cell mass and immediately observed the slides for bubble formation, to check for the presence of catalase.

β-hemolysis involves the total lysis of the red blood cells and is marked by the formation of a clear and transparent zone surrounding the colony on the agar plate, whereas α-hemolysis is marked by a distinct greenish zone around the colonies. We inoculated the MGC42 strain on nutrient agar plate supplemented with 5% sheep blood and incubated the plate at 37°C under aerobic conditions for 16–18 h. We observed the results under a bright light. The plates used were ready-made and were procured from HiMedia Laboratories Pvt. Ltd.

## Result and Discussion

### Identification Anomaly, Antibiogram Profiling, and Diagnostic Prospect

In the present study, we recovered the strain *Chryseobacterium gallinarum* MGC42 from the urine sample of a 20-year-old pregnant outpatient diagnosed with suspected UTI at the tertiary care hospital of our university. This isolate was also characterized by distinct bright yellow–colored colonies. The colonies displayed a drastic shift from their characteristic yellow color to brown when flooded with 20% KOH, suggesting that this coloration may have been imparted by the secretion of flexirubin type of pigment (**Supplementary Figure S1**) (Weeks, 1981).

This isolate was identified by the VITEK-II automated system (BioMérieux, France) as C. Indologenes. However, to our surprise, this strain was later reidentified as C. Gallinarum by amplification and sequencing of the 16S rRNA gene. Lin et al., 2017 detected a very low concordance between automated bacterial identification systems like VITEK-II, VITEK MS and molecular typing methods like 16S rRNA gene sequencing for the identification of Chryseobacterium species. This probably explains the identification anomaly in our study. The inability of such a system to distinguish between C. gallinarum and C. indologenes may create a false impression suggesting that the prevalence of C. gallinarum is low. Hence, there is every possibility that the prevalence of C. gallinarum may have been underestimated. Therefore, we propose the identification of Chryseobacterium species by automated systems like VITEK-II and VITEK MS that are over-reliant on factory default databases and often lack timely amendment must always be supplemented with 16S rRNA gene sequencing to confirm their identity which in turn will help guide proper therapeutic decisions.

The strain MGC42 was also resistant to several classes of antibiotics including penicillin, carbapenems, aminoglycoside, tetracycline, and polypeptide/polymyxin. Nevertheless, *Chryseobacterium* species have also been documented to be susceptible to some antibiotics. In our study, the strain MGC42 was similarly found to be susceptible to fluoroquinolones, sulphonamides, and tetracycline. To the best of our knowledge, the antibiotic susceptibility pattern of *C. gallinarum* has never been studied before. The antibiotic susceptibility pattern of the MICs of colistin and meropenem was ≥1,024 and ≥16 µg/ml, respectively, which agreed with the antibiotic susceptibility results derived from VITEK 2 (**Table 1**). This is particularly worrying as carbapenems and polymyxins are often the last viable options for the treatment of gram-negative bacterial infections (Armstrong et al., 2021; Mohapatra et al., 2021).

**Table 1:** Antimicrobial susceptibility testing using VITEK 2 system and micro-broth dilution–based MIC and MBC of *C. gallinarum* MGC42.

Group of Antibiotic/Drug Class	Antimicrobial	MIC (VITEK 2)	Interpretation	MIC (MBD)	MBC
Aminoglycoside	Amikacin	≥64	R	NT	NT
	Gentamicin	≥16			
	Netilmicin	≥32			
Carbapenems	Imipenem	≥16	R		
	Meropenem	≥16		≥16	32
Cephalosporins, Third Generation	Ceftazidime	ND		NT	NT
	Cefoperazone-Sulbactam	32	I		
Cephalosporins, Fourth Generation	Cefepime	ND			
Fluoroquinolone	Ciprofloxacin	1	S		
	Levofloxacin	1			
Glycylcycline	Tigecycline	≥8	R		
Penicillins	Ticarcillin-Clavulanic Acid	≥128	R		
	Piperacillin-Tazobactam	≥128			
Phosphonic	Fosfomycin	ND			
Polymyxin/Polypeptide	Colistin	≥16	R	≥1024	>1024
Sulphonamide	Trimethoprim-Sulfamethoxazole	≤20	S	NT	NT
Tetracycline	Minocycline	≤1	S		

*R, resistant; I, intermediate; S, sensitive; ND, not determined; NT, not tested.

### General and Specific Features of the *C. gallinarum* MGC42 Genome

**Figure 1:**
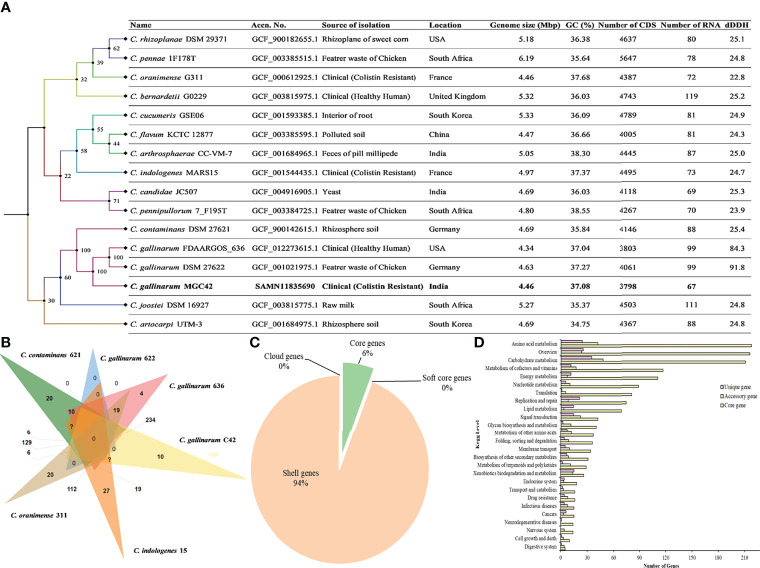
**(A)** Whole-genome–based phylogenetic tree between *C. gallinarum* MGC42 and other *Chryseobacterium* strain. Sequence highlighted the position of *C. gallinarum* MGC42 with their metadata. Phylogenetic inferences were obtained through TYGS tools. The branch color represents the bootstrap value. The branches of the tree are indicated by the genus and species name with type strains followed by other metadata of the respective species. **(B)** Venn diagrams generated by OrthoVenn show the distribution of shared and unique genes among six different sets of *Chryseobacterium* spp. **(C)** Core and accessory genes of *C. gallinarum* strains. **(D)** The numbers of core, cloud, soft, and shell genes were calculated by Roary. The core genes are shared by all the included organisms in the pangenome analysis.

A total of 6,557,593 paired reads were quality-filtered with 37.9% GC content. Furthermore, after assembly and reorientation, we obtained the size of the *C. gallinarum* MGC42 genome to be 4,349,499 bp with 96.67% genome coverage and 37.08% GC content and divided over into 61 contigs. All the contigs belong to the chromosomal DNA as no plasmid was detected (**Supplementary Table S1**). The Type (Strain) Genome Server (TYGS) returned *C. gallinarum* DSM 27622 as the closest type with 98% similarity (**Figure 1A**). The Prokka predicted a total of 3,879 ORFs, which includes 3,799 protein-coding ORFs and 80 RNAs (three rRNAs, 63 tRNAs, one tmRNAs, and 13 misc_RNAs). Of the 3,879 ORFs, 1,911 (49.26%) were assigned a putative function, whereas 1,968 (50.73%) were annotated as hypothetical proteins.

A total of 1,246 unique K numbers were assigned to 1,531 (39.46%) ORFs, which are further mapped to 240 different KEGG pathways based on the scoring criteria (**Supplementary Table S2**). The top mapped pathways were metabolic pathways (462), biosynthesis of secondary metabolites (220), microbial metabolism in diverse environments (120), biosynthesis of cofactors (105), and carbon metabolism (72). The pathways for metabolism in diverse envirnoments were ranked third in genome of MGC42 strain. This finding corroborates with the fact that Chryseobacterium species are ubiquitously distributed in the natural environment (Bernardet et al., 2006).

Using the combination of AMR databases, k-mer, and a machine learning–based classifier–based improved annotation, we identified the 12 antimicrobial resistance (AMR) genes belonging to resistance mechanism like antibiotic efflux (6), inactivation (3), target alteration (3), and target replacement (1) including two putatively novel β-lactamases (**Supplementary Table S3**). Our findings show that the AMR gene identification is consistent with the antibiotic resistance profile of MGC42. We also screened the homologs of the genes commonly associated with colistin resistance (*pho*P, ORF01530; *pho*Q, ORF00881; *pmr*A, ORF00440; *pmr*B, ORF00439; *lpx*A, ORF00011; *lpx*C, ORF00012; *lpx*D, ORF00013) for mutation analysis. Our study revealed that only *pmr*B and *lpx*D harbor single-point mutation. The two-component protein *pmr*B harbors a single-point mutation at position 384 (M384T). Similarly, the third enzyme of the LPS biosynthesis pathway (*lpx*D) harbors a single-point mutation at position 66 (I66V). These mutations could most likely be responsible for colistin resistance in *C. gallinarum* MGC42 (**Supplementary Figure S2**).

Three-hundred forty genes are predicted as a secreted and non-secreted virulence factors (VFs) (**Supplementary Table S4**). Most ORFs belonged to the type I secretion system (T1SS), type VI secretion system (T6SS), bacterial secretion system (Sec), and quorum sensing (QS) categories of VFs (**Table 2**). To the best of our knowledge, virulence profile of *C. gallinarum* has never been studied before. These predicted VFs further need to be classified into different VF categories, which need deep-annotation and further experimental validations.

**Table 2:** List of predicted virulence factors and their respective VF classes.

VF Class	Subclass	ORF	KO Number	Gene	Description
Type I secretion system	ABC transporters	ORF00074	–	hlyD	Hemolysin secretion protein d
		ORF03531	K02065	MetN	Methionine import atp-binding protein
		ORF00740	K02066	MlaE	Intermembrane phospholipid transport system permease protein
		ORF00727	K02067	hp	Hypothetical protein
		ORF03531	K02071	MetN	Methionine import atp-binding protein
		ORF03532	K02072	MetI	D-methionine transport system permease protein
		ORF03533	K02073	MetQ	D-methionine-binding lipoprotein
		ORF00001	K06861	LptB	Lipopolysaccharide export system atp-binding protein
		ORF02984	K07091	hp	Hypothetical protein
		ORF01293	K09690	hp	Hypothetical protein
		ORF01292	K09691	TagH	Teichoic acids export atp-binding protein
		ORF03745	K09808	LolE	Lipoprotein-releasing system transmembrane protein
		ORF02767	K09810	LolD	Lipoprotein-releasing system atp-binding protein
		ORF02242	K09811	FtsX	Cell division protein
		ORF00530	K09812	FtsE	Cell division atp-binding protein
		ORF01202	K11720	hp	Hypothetical protein
		ORF00500	K18889	YheI	Putative multidrug resistance abc transporter atp-binding/permease protein
		ORF00396	K18890	NA	Putative abc transporter atp-binding protein
		ORF00500	K18889	YheI	Putative multidrug resistance abc transporter atp-binding/permease protein
		ORF00396	K18890	NA	Putative abc transporter atp-binding protein
Type VI secretion system		ORF01931	–	vgrG1a	NA
		ORF02111	–	vgrG1c	NA
		ORF01506	–	Hp	NA
		ORF02959	–	vgrG1a	NA
		ORF03302	–	Hp	NA
Bacterial secretion system (Sec)		ORF00833	K03070	SecA	Preprotein translocase subunit
		ORF00433	K03073	SecE	Preprotein translocase subunit
		ORF01866	K03075	SecG	Preprotein translocase subunit
		ORF01412	K03076	SecY	Preprotein translocase subunit
		ORF03130	K03106	SRP54	Signal recognition particle subunit
		ORF00194	K03110	ftsY	Fused signal recognition particle receptor
		ORF01523, ORF02447	K03116	TatA	Sec-independent protein translocase protein
		ORF02447	K03117	TatB	Sec-independent protein translocase protein
		ORF03209	K03118	TatC	Sec-independent protein translocase protein
		ORF00497	K03210	YajC	Preprotein translocase subunit
		ORF00344	K03217	YidC/Oxa1	Yidc/oxa1 family membrane protein insertase
		ORF01506, ORF01931, ORF02111, ORF02959, ORF03302	K11904	NA	T6ss
		ORF02441	K12257	SecD/SecF	Secd/secf fusion protein
		ORF00982, ORF03199, ORF03646	K12340	NA	Outer membrane protein
Quorum sensing		ORF00084	K20483	nisB	Nisin biosynthesis protein nisb
		ORF00085	K20483	Hp	Hypothetical protein
		ORF00194	K03110	ftsY	Signal recognition particle receptor ftsy
		ORF00227	K18139	oprM	Outer membrane protein oprm
		ORF00344	K03217	yidC2	Membrane protein insertase yidc 2
		ORF00433	K03073	Hp	Hypothetical protein
		ORF00497	K03210	yajC	Sec translocon accessory complex subunit yajc
		ORF00604	K01114	plcN	Non-hemolytic phospholipase c
		ORF00833	K03070	secA	Protein translocase subunit seca
		ORF01052	K18139	oprM	Outer membrane protein oprm
		ORF01412	K03076	secY	Protein translocase subunit secy
		ORF01520	K01497	ribA	Gtp cyclohydrolase-2
		ORF01675	K11752	ribD	Riboflavin biosynthesis protein ribd
		ORF01820	K18139	oprM	Outer membrane protein oprm
		ORF01866	K03075	Hp	Hypothetical protein
		ORF02228	K01897	FadD15	Long-chain-fatty-acid–coa ligase fadd15
		ORF02239	K20276	Hp	Hypothetical protein
		ORF02328	K01658	pabA	Aminodeoxychorismate synthase component 2
		ORF02329	K01657	trpE	Anthranilate synthase component 1
		ORF02441	K12257	secDF	Protein translocase subunit secdf
		ORF02582	K15657	srfAD	Surfactin synthase thioesterase subunit
		ORF02628	K13075	NA	Putative metallo-hydrolase
		ORF02964	K18139	oprM	Outer membrane protein oprm
		ORF03078	K20483	Hp	Hypothetical protein
		ORF03079	K20483	nisB	Nisin biosynthesis protein nisb
		ORF03080	K20484	Hp	Hypothetical protein
		ORF03130	K03106	ffh	Signal recognition particle protein
		ORF03458	K06998	yddE	Putative isomerase ydde
		ORF03678	K01897	FadD15	Long-chain-fatty-acid–coa ligase fadd15

While understanding the pathogenicity of a bacterium, toxins including neurotoxin play a key role in causing severe human ailments (Mansfield et al., 2019). Therefore, to answer this, we also explored the genome of the strain MGC42 using a state-of-the-art approach as to date, no experimental and sequencing information about the toxicity of this species is available. In this regard, we predicted 28 putative secretory and non-secretory toxins. Apart from this, we predicted 39 uncharacterized ORFs, which could potentially belong to a novel toxin family and might be species-specific, which needs further *in silico* and *in vitro* characterization (**Supplementary Table S5**).

The cluster of orthologous gene of the strain MGC42 was compared with *C. gallinarum* DSM 27622, *C. gallinarum* FDAARGOS_636, *C. contaminans* DSM 27621, *C. oranimense* G311, and *C. indologenes* MARS15 to provide insights into conserved cellular components, biological processes, and molecular functions. It was found that among 4,277 clusters, 3,080 orthologous clusters contain at least three species, 1,368 orthologous clusters contains at least two species, and 1,482 singletons. Of this, 78 essentially *paralogous* genes (*in-paralogs*) were predicted in *C. gallinarum* MGC42 that could be due to the divergence of lineages and duplication within the lineage of *C. gallinarum* species (**Figure 1B**).

The prediction of conserved homologous/orthologous was further improved by performing core-genome analysis in between all six species using Roary. The core-genome analysis genomes revealed that they all shared 683 genes (6%) in the core region of their genomes, whereas 11,120 genes (94%) are found as accessory/shell genes. The majority (~73%) of accessory genes were strain-specific having a specific role to interact with the host or helping in niche adaptation (**Figure 1C**). Among them, 2,427 genes are shared by *C. gallinarum* DSM 27622 (Chicken feathers), *C. gallinarum* FDAARGOS_636 (Healthy Human), and *C. gallinarum* MGC42 (patient with UTI). Moreover, we also identified a novel toxin Ntox23 domain (PF15528) containing proteins (ORF01631 and ORF00315) of polymorphic toxin systems with conserved ND, DxxR motifs, and a histidine residue, which is exported by TcdB/TcaC secretion system in the strain MGC42 (**Supplementary Table S5**) (Zhang et al., 2012). We hypothesize that this additional VF identified only in the strain MGC42 might have a role to play in its ability to colonize and infect human host. At functional level, the core genes were mostly associated with amino acid metabolism, carbohydrate metabolism, metabolism of cofactors and vitamins, energy metabolism, nucleotide metabolism, and translation. However, the unique and accessory genes have higher proportion of carbohydrate metabolism, amino acid metabolism, signal transduction, metabolism of cofactors and vitamins, xenobiotics biodegradation, and metabolism categories (**Figure 1D**, **Supplementary Figure S3**).

**Figure 2:**
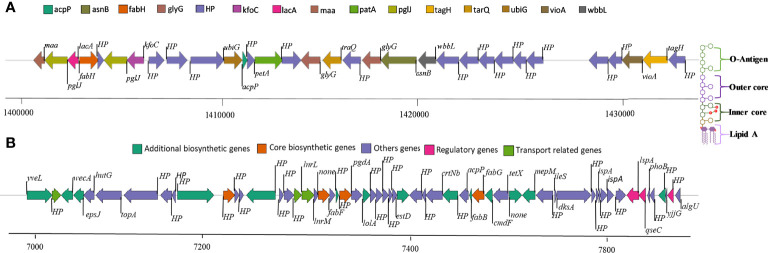
**(A)** O-antigen synthase operon arrangement in *C. gallinarum* MGC42 starting from 1411059 to 1420661 bp. **(B)** Polyketide synthesis operon arrangement in *C. gallinarum* MGC42 at ORF 700929 to 765695. Genes of the same functional classification are shown in the same color.

Genome mining through antiSMASH revealed distinctive secondary metabolite clusters, like resorcinol/arylpolyene (felxirubin-like), non-ribosomal peptide synthetases (NRPS), lanthipeptide, microviridin, and siderophore (putrebactin/avaroferrin) biosynthetic gene clusters (BGCs). In contrast, we further explored the felxirubin-like pigment synthesis genes cluster that shared 75% similarity with known flexirubin biosynthesis cluster (BGC0000838), indicating that it is most likely novel and species-specific (**Figure 2B**). Furthermore, we also located the genes cluster of o-antigen (lipopolysaccharide biosynthesis) as shown in **Figure 2A**, whereas the genes of k-antigen cluster (CPS biosynthesis) as they are not in continues sequence in the genome and could be due the unfinished draft genome (**Supplementary Table S6**). The phage-driven gene flow analysis using the online server PHASTER revealed five different incomplete phage-like regions encoded the Phage-like Protein, Coat Protein, Tail Shaft, Fiber Protein, Terminase, and portal protein as shown in the **Supplementary Figure S4**.

### Identification of novel of β-Lactamase Genes

We identified an 879-bp ORF (ORF03368) encoding a protein having 292–amino acid residues sharing 73.29% amino acid identity with *bla*_CIA-4_, an extended-spectrum β-lactamase encoded on a 143.85-kb contig (**Supplementary Figure S5A**). A serine active site characteristic of β-Lactamases was found within this mature protein sequence. It has all the four conserved elements of class A β-Lactamases: a Ser-X-X-Lys consensus active site serine residue at position 70 (Fan et al., 2007), an SDN loop at position 130, a conserved Glu166, and a KTG sequence at position 234. This distantly related Ambler class A *bla*_CIA-like_ gene has a G+C content of 41% and is most likely responsible for β-lactam antibiotics resistance in this organism. We identified another 723-bp ORF (ORF03015) encoding a 240–amino acid residue protein in a 203.52-kb contig. It shares 82.92% amino acid identity with *bla*_CGB-1_, a class B β-lactamase and is most likely responsible for resistance to imipenem (**Supplementary Figure S5B**). Both genes are most likely novel and require further biochemical characterization. These AR genes were also flanked by many hypothetical proteins with no BLAST hit. There might be a possibility that those sequences carry transposases and unknown insertion elements responsible for the mobilization of these genes.

### Pathogenic Characterization of MGC42

The TEM of *C. gallinarum* MGC42 strain in presence of negatively stained CsCl revealed the presence of secreted capsular material (**Supplementary Figure S6**). From genome analysis, we located 21 homologous ORFs related to CPS biosynthesis in a different region of the first contigs and could be a putatively novel cluster specific to this species (**Supplementary Table S6**). The capsular material that is anchored on the outermost layer of the cell is often involved in mediating direct interactions between the bacteria and its environment and is therefore considered an important VF for many bacterial pathogens (Taylor and Roberts, 2004). Apart from pathogenicity due to CPS, this could be one of the reasons for contributing toward colistin resistance by MG42 strain that is further supported by the report on colistin resistance mechanism acquired by *K. pneumoniae* due to the presence of secreted capsular material (Campos et al., 2004).

**Figure 3:**
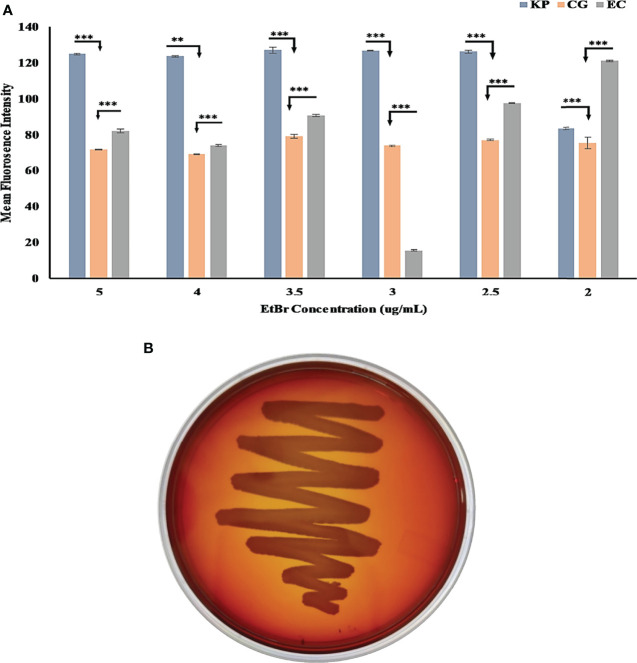
**(A)** Bar graph of *C. gallinarum* C42 and *K. pneumoniae* SDL79 strains along with *E. coli* ATCC 25922 by the EtBr agar method. The error bar represent the mean standard error, whereas the star represents the significant difference in efflux activity between pair of SDL79 - MGC42 and ATCC 25922 - MGC42 at different p-value (* <0.05; ** <0.01; *** <0.001). The x-axis represents the concentration of EtBr, and the y-axis represents the mean fluorescence intensity area value. **(B)** *C. gallinarum* MGC42 showing α-hemolysis in greenish color on 5% sheep blood agar plates. Culture was incubated at 37°C for 18 ± 2 h.

The MGC42 strain showed a moderate efflux activity in all concentrations of ethidium bromide (EtBr) starting from 2.0 to 5.0 µg/ml (**Figure 3A**) in comparison with *E. coli* ATCC 25922 (non-pathogenic) and *K. pneumoniae* SDL79 (pathogenic) (Dey et al., 2020). As this strain has moderate efflux activity, we further improved the annotation of ORFs using the KEGG database. A total of 110 ORFs were assigned with K numbers through KEGG mapping and classified into 14 unique superclasses of transporter genes and might be involved in pathogenicity in many different ways. It includes ABC-2 type, drug aquaporins, organic acid protein small neutral solute, metallic cation, iron-siderophore, vitamin B12, saccharide, polyol, lipid, phosphate, amino acid, and unknown transporter superclasses (**Supplementary Table S7**). In addition to their role in resistance mechanism, it is proven that efflux pumps are key factors involved in the detoxification of intracellular metabolites, exporting VFs, biofilm formation, pH homeostasis, and QS (Martinez et al., 2009; Teelucksingh et al., 2020; Alav et al., 2021). However, to date, no documental evidence is reported that explains the role of efflux pumps in this species. These transporters might have a role to play in this strain’s niche adaptation.

Catalase is one of the key antioxidant enzymes, and it appears to be involved in protection against immune infection and oxidative stress. The excessive hydrogen peroxide (H_2_O_2_) produced by the host defense response is a poisonous compound to bacteria and acts as a second messenger in signal transduction pathways. At the genomic level, the hydrogen peroxide–inducible gene activator (*Oxy*R: ORF00677) and a LysR family transcriptional regulator are involved in the hydrogen peroxide (H_2_O_2_) defense mechanism through the activation of the catalase enzymes (Parvatiyar et al., 2000). The presence of ORFs for catalase-peroxidase (*kat*G: ORF0165 and *kat*E: ORF2808) enzymes in the genome of the MGC42 strain is known to reduce H_2_O_2_ to water and oxidize it to molecular oxygen (Zamocky et al., 2008). This was further supported by glass slide–based catalase test, where upon the addition of three to four drops of 3% medical H_2_O_2_, the MGC42 strain immediately exhibited bubbling effect due to breakdown of H_2_O_2_ into water and oxygen (**Supplementary Figure S7**). However, the non-pathogenic *E. coli* ATCC 25922 strain, which is taken as negative control, ensures no production of oxygen bubbles.

Species from the genus *Chryseobacterium* have shown no-hemolysis to strong hemolysis activity (Hsueh et al., 1996; Mehta and Pathak, 2018). When the MGC42 strain was grown on 5% sheep blood agar nutrient media for 16 to 18 h, the hemoglobin of blood cells around and below the colonies was reduced to green methemoglobin, which was confirmed by the strain’s α-hemolytic activity (**Figure 3B**). The oxidation of oxy-hemoglobin (Fe+2) to non-oxygen-binding methemoglobin (Fe+3) by hydrogen peroxide causes α-hemolysis (McDevitt et al., 2020). The *hly*D (ORF00074) protein, the second component of the hemolysin secretion system (T1SS), is responsible for this phenomenon; however, we did not find any other component of this system in the genome of the MGC42 strain.

### Transition From the Environment to Human

This study provides insight into changes in major genomic features, specifically unique genes, indicating the uncommon potential of a new clinically identified bacterium *C. gallinarum* MGC42 isolated initially from the pharyngeal scrape of a health chicken (Kämpfer et al., 2014).

Unravelling the regulatory systems that govern bacterial pathogens’ transition from a free-living non-pathogenic state to a virulent state will be the next critical step toward understanding *C. gallinarum* to establish strategies for regulating its spread. Similarly, the signaling pathways that may have driven the formation of its multicellular communities in actual hosts must be determined in the future. The *C. gallinarum* MGC42 could be used as a model organism to understand their diagnosis, the genetic basis of disease, and treatment that could take a giant leap forward with the creation of networks to connect clinicians with geneticists and their lifestyle switching. In addition, this bacterium could be an opportunistic human pathogen in immunocompromised patients in future.

Uncharacterized VFs, mechanisms of pathogenesis, and the absence of ecological and epidemiological knowledge compounded by existing resistance to several classes of antibiotics could make treatment of *C. gallinarum* infection challenging. Moreover, the latest VITEK-GNI card’s failure to distinguish this organism from *C. indologenes* indicates that its prevalence in the hospital may have been significantly undermined. Our finding warrants the implementation of molecular typing to direct appropriate antibiotic regimens without over-reliance on the VITEK 2 system that utilizes the factory default database lacking in the timely amendment. Therefore, we recommend more prolonged periods of laboratory-based surveillance with population-based data to determine the prevalence of *C. gallinarum* infections. To the best of our knowledge, this is the first documented evidence of the emergence of *Chryseobacterium gallinarum* as a human pathogen.*in vitro*

## Data Availability Statement

The whole-genome sequence of the C. gallinarum MGC42 has been deposited in NCBI under the GenBank assembly accession: GCA_023604885.1.

## Authors Contributions

MG, SDe, AS and ES: Conceptualization; MG, SDe, AS, SS, and JZ: Investigation; MG, SDe, SDi, and DB: Formal analysis; MG, SDi, and SS: Visualization; MG, SDe, AS, RS, and ES: Writing-original draft; All author: Writing-review and editing; ES: Funding acquisition; M and ES: Project administration and supervision. All authors contributed to the article and approved the submitted version.

## Funding

This work is supported by ICMR, New Delhi (Grant No. OMI-Fellowship/1/2018-ECD-I) and partially supported by SERB, New Delhi (Grant No. EMR/2016/006732).

## Conflict of Interest

The authors declare that the research was conducted in the absence of any commercial or financial relationships that could be construed as a potential conflict of interest.

## Publisher’s Note

All claims expressed in this article are solely those of the authors and do not necessarily represent those of their affiliated organizations, or those of the publisher, the editors and the reviewers. Any product that may be evaluated in this article, or claim that may be made by its manufacturer, is not guaranteed or endorsed by the publisher.
