# Supplementary material for: Characterization and Comparative Genomic Analysis of a Highly Colistin-Resistant Chryseobacterium gallinarum: a Rare, Uncommon Pathogen
Source: Front Cell Infect Microbiol. 2022 Jul 14;12:933006. doi: 10.3389/fcimb.2022.933006 (PMC9329510; doi:10.3389/fcimb.2022.933006)
Supplement: Supplementary file 1 [file DataSheet_1.pdf]

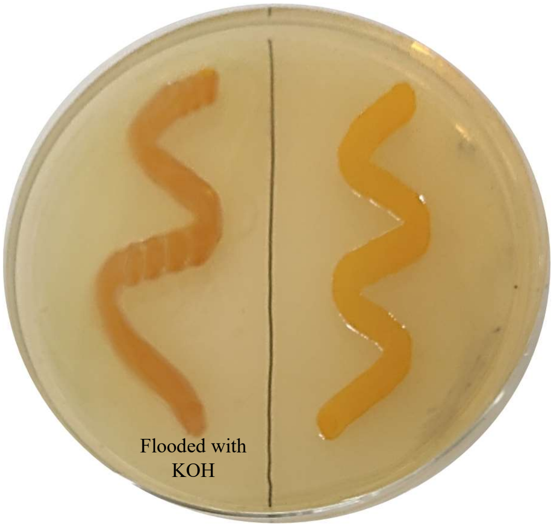

Supplementary Fig. S1: Shifting in colour of *C. gallinarum* MGC42 colony from muddy yellow to dark red/brown with addition of 20% KOH.

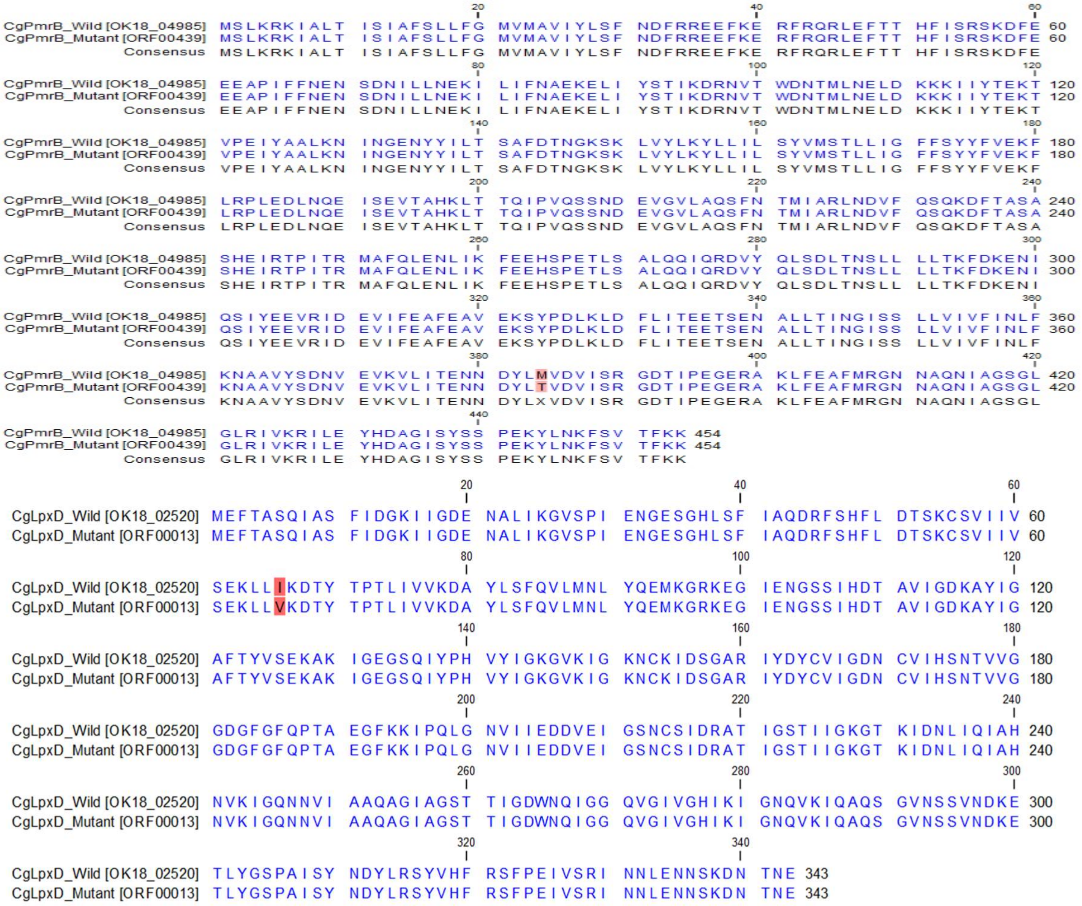

Supplementary Fig. S2: Pair-wise protein sequence alignment of: A) Mutant *pmrB protein* wild-type *lpx*D protein of *C. gallinarum* DSM 27622 (AKK72079.1). B) Mutant *lpxD* protein with wild-type *lpx*D protein of *C. gallinarum* DSM 27622 (AKK71662.1). The mutations is highlighted by orange coloured box.

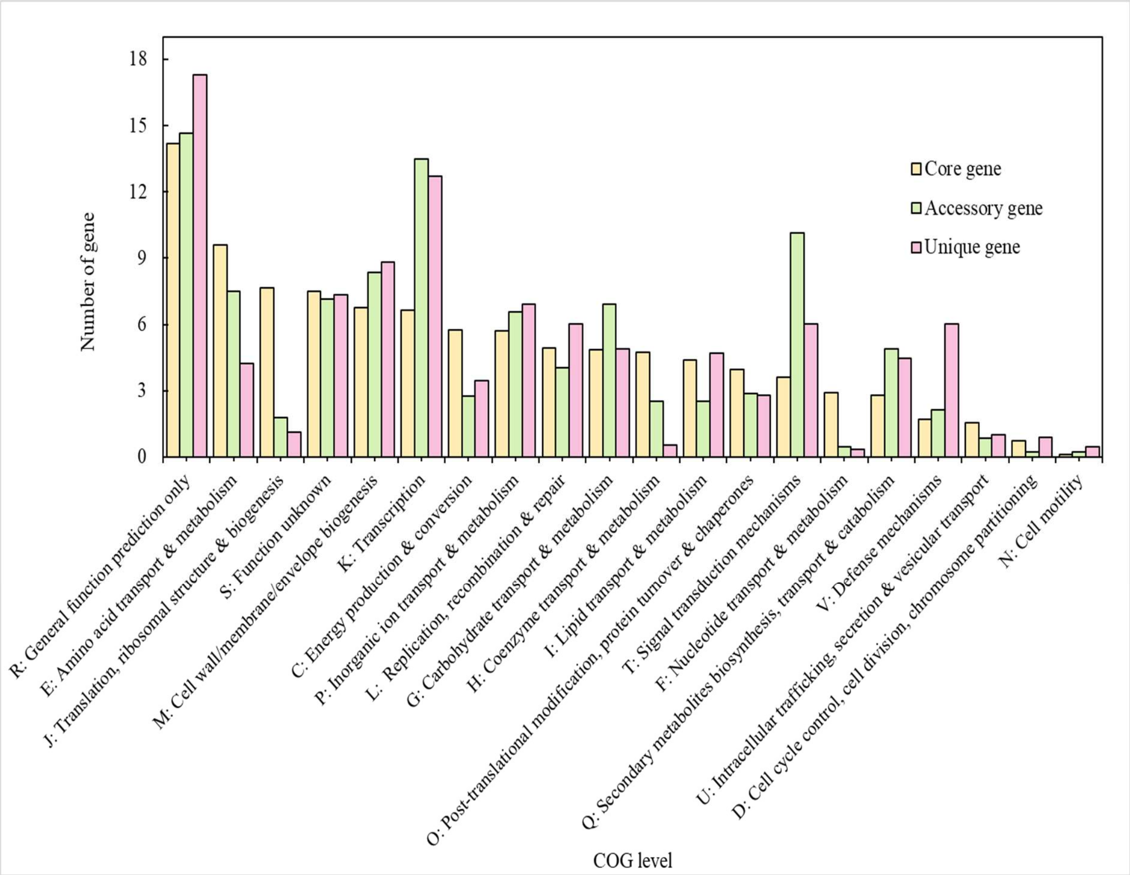

Supplementary Fig. S3: COG distribution of core, accessory and unique genes present in the *C. gallinarum* MGC42 genomes generated from pangenome analysis.

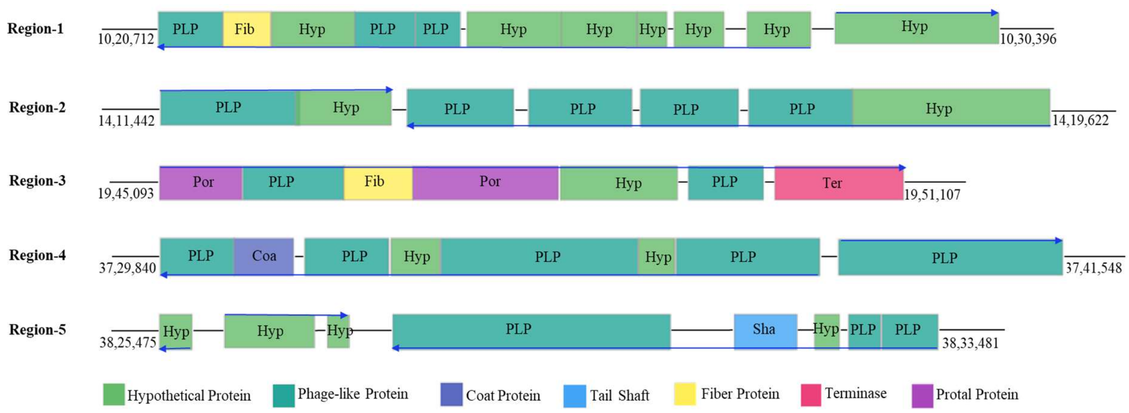

Supplementary Fig. S4: Schematic Island of 5 prophage incomplete regions bearing by *C. gallinartum* MGC42 its chromosome. The number represents the start and end position of the respective region in the genome. The different protein types were represent in different colour according to their functions. The result is obtained with PHASTER.

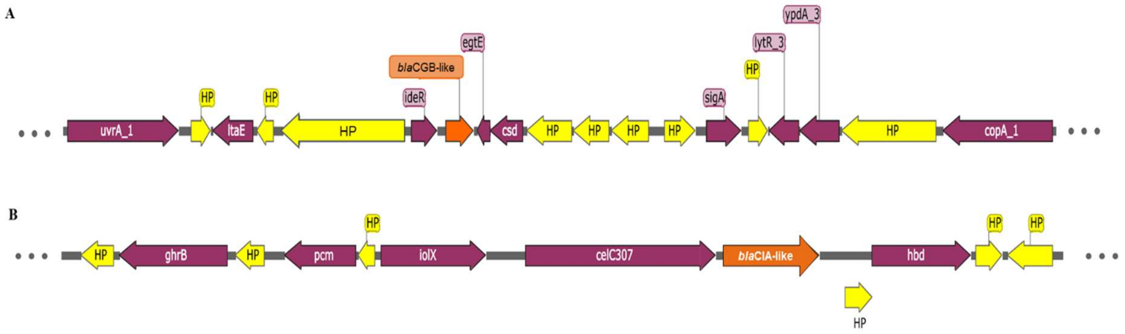

Supplementary Fig. S5: Schematic Island of **(A)** *bla*$_{\text{CGB-like}}$ gene of 240 aa length and **(B)** *bla*$_{\text{CIA-like}}$ gene of 292 aa length. The genes of interest mentioned above shown with different coloured arrows. ORFs annotated with a putative function is shown as maroon-coloured arrows. The yellow coloured arrows represent other ORFs that translated into hypothetical proteins (HPs) with no BLAST hits. These predicted HPs flanking the AR genes might be probable carriers of transposons or unknown insertion elements by which the bacterium might have acquired both the beta-lactamase genes. The illustrations were created using the SnapGene.

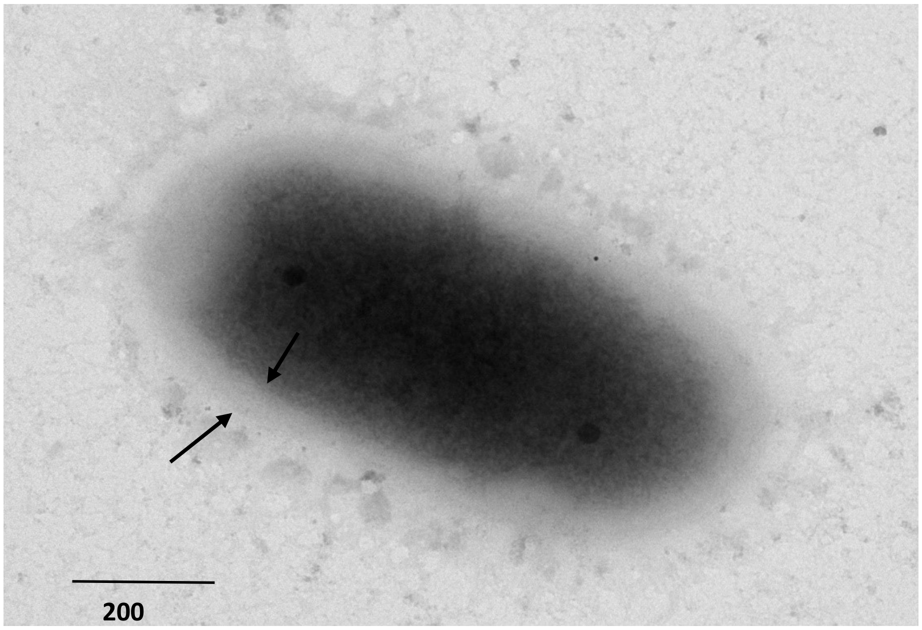

Supplementary Fig. S6: Transmission electron microscopic image of *C. Gallinarum* MGC42 at an operating voltage of 200 k. The light area between two black arrows is the secreted capsular polysaccharide.

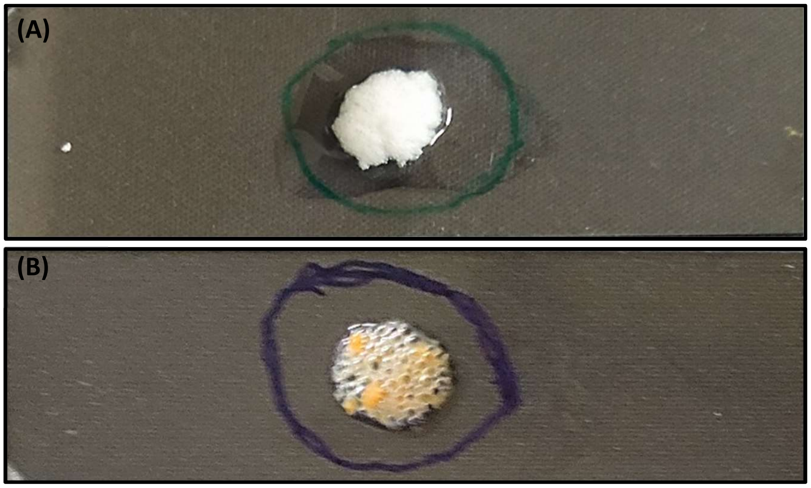

Supplementary Fig. S7: Catalase reaction for (A) *E. coli* ATCC 25922 and (B) *C. gallinartum* MGC42. The Catalase Test is used to detect whether or not an unknown bacterium has the enzyme, catalase. Bubbling appears as catalase enzyme was produced by the bacterial species due to breakdown of hydrogen peroxide (H_2O_2) into water molecule and elemental oxygen. Reagent used- 3% H_2O_2, Trypticase Soya Agar.
